# Ah receptor, vitamin B12 and itaconate: how localized decrease of vitamin B12 prevents survival of macrophage-ingested bacteria

**DOI:** 10.3389/ftox.2024.1491184

**Published:** 2024-12-11

**Authors:** Karl Walter Bock

**Affiliations:** Institute of Experimental and Clinical Pharmacology, Tübingen, Germany

**Keywords:** Ah receptor, vitamin B12, itaconate, macrophages, bacteria

## 1 Introduction

Ah receptor (AHR) is a ligand-dependent transcription factor and environmental sensor (Poland et al., 1976; Gu et al., 2000). The receptor has been discovered in studies of toxicity of the persistent AHR ligand TCDD (2,3,7,8-tetrachlorodibenzo-p-dioxin) leading to deregulation of the receptor. Recently, a number of physiological receptor functions have been identified. One of its multiple physiological functions is involved in intestinal barrier integrity and host-microbiome interaction (Stockinger et al., 2021; Bock, 2020). The microbiome of human colon includes anaerobic archaea and bacteria generating vitamin B12 (Mahammadzadeh et al., 2022; Sokolovskaya et al., 2020). Surprisingly, vitamin B12 has been recently identified as natural AHR antagonist (Kim et al., 2020). This important finding appears to be unrecognized by many scientists in the AHR field. A previous minireview (Bock, 2023) and the present opinion on links between Ah receptor, vitamin B12 and itaconate are intended to stimulate the interest in the unsolved finding that vitamin B12 is a direct antagonist of the AHR.

## 2 Links between Ah receptor, vitamin B12 and itaconate

### 2.1 Vitamin B12 and folic acid as natural AHR antagonists

Both vitamin B12 and folic acid have been identified as natural antagonists of the ligand-modulated AHR (Kim et al., 2020). Indead, the two vitamins directly bind to the receptor in the cytosol, blocking AHR’s nuclear translocation and transcriptional signaling. In this way, they possibly compete with many environmental toxic ligands such as TCDD, dietary phytochemicals and microbial products including indoles and pigmented virulence factors (Moura-Alves et al., 2014). Vitamin B12 and folic acid are known to be cofactors of the enzyme methionine synthase in the one-carbon cycle facilitating the *de novo* synthesis of nucleotides and methylation of DNA and protein (Ducker and Rabinowitz, 2017). Here, the focus is on B12-dependent methylmalonyl-CoA mutase (MUT) that is involved in lipid metabolism, particularly in converting propionyl-CoA to the TCA cycle metabolite succinyl-CoA. However, the second vitamin B12-dependent enzyme, methionine synthase, should not be neglected in studies of possible links between AHR and vitamin B12, as discussed in section 3.

### 2.2 Decreased vitamin B12 due to generation of toxic itaconyl-CoA in mitochondria

It has been demonstrated that macrophages activated by microbial pathogen-associated molecular patterns and cytokines upregulate expression of the enzyme cis-aconitate decarboxylase (CAD), also termed ACOD1 and Irg1, generating the antimicrobial and antiinflammatory itaconate (Michelucci et al., 2013; Lang and Siddique, 2024). CAD is induced by the key antioxidant regulator Nrf2 (Mills et al., 2018) and by AHR since Nrf2 has been demonstrated to operate in mutual crosstalk with the AHR (Miao et al., 2005; Shin et al., 2007). Increased itaconate levels in mitochondria lead to toxic itaconyl-CoA that has been demonstrated to form a stable biradical when bound to the enzyme MUT leading to decreased vitamin B12 (Shen et al., 2017; Ruetz et al., 2019).

The toxic reaction of itaconyl-CoA with MUT is supported by studies of TCDD-treated mice. These studies demonstrated that B12-dependent MUT activity is inhibited in mitochondria, leading to decreased vitamin B12 serum levels thereby redirecting propionyl-CoA metabolism to an alternative ß-oxidation-like pathway resulting in acrylyl-CoA conjugate buildup leading to non-alcoholic fatty liver (NAFLD) (Orlowska et al., 2022).

### 2.3 Decreased vitamin B12 leading to death of macrophage-ingested bacteria

**FIGURE 1:**
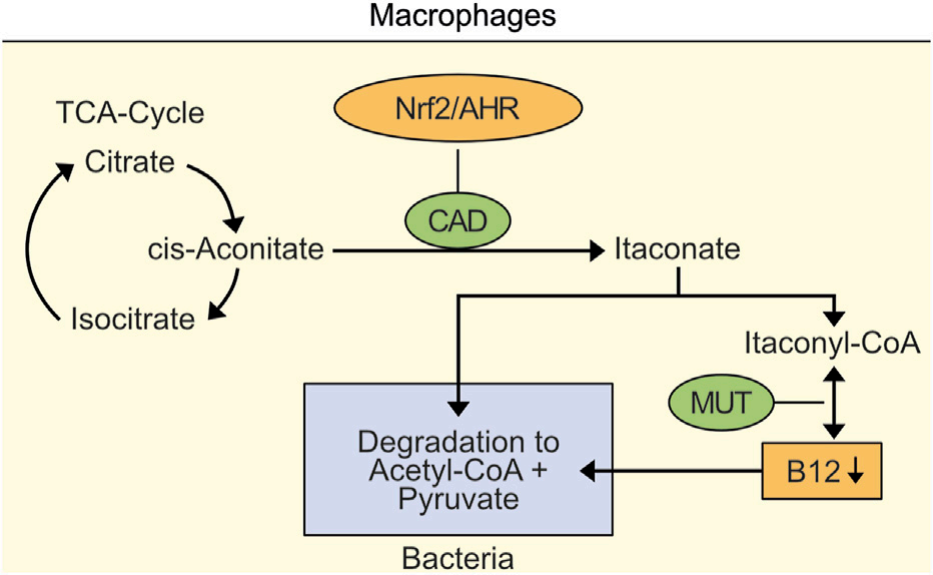
Localized decrease of vitamin B12 in macrophages as an example for inhibition of survival of macrophage-ingested bacteria. Nrf2- and AHR-induced cis-aconitate decarboxylase (CAD) leads to accumulation of itaconate and toxic itaconyl-CoA. Toxic Itaconyl-CoA has been demonstrated to decrease B12 levels by inhibiting methylmalonyl-CoA mutase (MUT). Importantly, decreased B12 levels inhibit survival of macrophase-ingested bacteria.

Importantly, decreased B12 levels in macrophages also inhibit survival of macrophage-ingested bacteria (Figure 1) (Ryan et al., 2020). This inhibition is necessary since bacteria such as *M. tuberculosis* managed to degrade itaconate (Sasikaran et al., 2014). Macrophages may temporarily tolerate a localized decrease of B12.

## 3 Links between AHR and vitamin B12 signalling in intestinal tissue repair

As mentioned in the introduction, AHR is involved in intestinal barrier integrity and host-microbiome interaction (Stockinger et al., 2021). In particular, AHR signalling is required for the resolution of injury-induced colonic stem cells (Sha et al., 2022). In this context it is interesting that vitamin B12 is a limiting factor for induced cellular plasticity and tissue repair. In the dextran sulfate sodium model of acute ulcerative colitis serum vitamin B12 was observed to be depleted and vitamin B12 supplementation enhanced specific markers of epigenetic histone methylation (Kovatcheva et al., 2023). Hence, there are links between AHR and vitamin B12 signaling in the intestine.

## 4 Discussion

It is understood that both AHR and vitamin B12 are involved in complex, tissue-dependent signalling networks. Tissue-dependent links between AHR and the two vitamin B12-dependet enzymes, methylmalonyl-CoA mutase (MUT) and methionine synthase have been discussed here. Notably, persistent AHR activation has to be avoided since it may lead to toxicity and cancer. In studies using a constitutively-active AHR mutant it has been demonstrated that the gastric mucosa is particularly affected (Dantsuka et al., 2019). Interestingly, the gastric pump inhibitor omeprazole has been identified as AHR agonist (Diaz et al., 1990). The discussed opinion deals with tissue-dependent links between AHR, vitamin B12 and itaconate, for example, with findings how the decrease of vitamin B12 leads to death of macrophage-ingested bacteria. It is hoped the discussion may stimulate the interest of AHR scientists in attempts to substantiate and understand the surprising finding that vitamin B12 is a direct antagonist of the AHR (Kim et al., 2020).
